# Exportations of Symptomatic Cases of MERS-CoV Infection to Countries outside the Middle East

**DOI:** 10.3201/eid2204.150976

**Published:** 2016-04

**Authors:** Cristina Carias, Justin J. O’Hagan, Amy Jewett, Manoj Gambhir, Nicole J. Cohen, Yoni Haber, Nicki Pesik, David L. Swerdlow

**Affiliations:** IHRC, Inc., Atlanta (C. Carias, J.J. O’Hagan);; Centers for Disease Control and Prevention, Atlanta, Georgia, USA (C. Carias, J.J. O’Hagan, A. Jewett, M. Gambhir, N.J. Cohen, Y. Haber, N. Pesik, D.L. Swerdlow);; Monash University, Melbourne, Victoria, Australia (M. Gambhir)

**Keywords:** Middle East, coronavirus, exportations, Middle East respiratory syndrome coronavirus, MERS-CoV, travel, disease spread, viruses

## Abstract

In 2012, an outbreak of infection with Middle East respiratory syndrome coronavirus (MERS-CoV), was detected in the Arabian Peninsula. Modeling can produce estimates of the expected annual number of symptomatic cases of MERS-CoV infection exported and the likelihood of exportation from source countries in the Middle East to countries outside the region.

In September 2012, the first confirmed case of Middle East respiratory syndrome coronavirus (MERS-CoV) infection was reported in the Kingdom of Saudi Arabia (KSA) (*1*). Approximately 900 laboratory-confirmed cases had been identified as of January 16, 2015; more than 300 (37%) have resulted in death. Although KSA has reported >80% of cases, infections have been confirmed in other Middle Eastern, European, North American, and, more recently, Asian countries (*2*). Multiple introductions from an animal reservoir have occurred (*3*), but human-to-human transmission of MERS-CoV has also been documented among family members of case-patients and healthcare personnel who cared for case-patients (*4*–*6*). More than a dozen cases have been identified among travelers returning from the Middle East to their home countries; onward transmission in destination countries also occurred (*7*,*8*).

## The Study

We calculated the expected annual number of exportations of symptomatic cases of infection with MERS-CoV and the likelihood of exportation from the Middle Eastern countries where additional cases have been detected (KSA, Jordan, Qatar, and the United Arab Emirates [UAE], which we refer to as source countries) to countries outside the Middle East. We defined exportation as the arrival of a person infected with MERS-CoV in a country other than KSA, Jordan, Qatar, and UAE as a result of MERS-CoV infection in those source countries and subsequent outbound travel (not including medical evacuation). Because it is unclear whether MERS-CoV cases in Middle Eastern countries other than the source countries resulted from importation or a local outbreak, those countries were excluded from the analysis. Exportations can occur by visitors returning to their home countries or source country residents traveling abroad. We produced example calculations for destination countries outside the Middle East where exportation of MERS-CoV infection cases has been confirmed (Algeria, Austria, France, Greece, Italy, Malaysia, Netherlands, Tunisia, United Kingdom, and the United States) as of January 16, 2015 (Table 1).

**Table 1:** Number of symptomatic cases of MERS-CoV infection and number of symptomatic cases exported to countries outside the Middle East, overall and during January 1, 2013–January 16, 2015*

Region and country	No. cases overall (no. cases Jan 1, 2013–Jan 16, 2015)
Source countries	
KSA	831 (826)
UAE	67 (67)
Jordan	9 (7)
Qatar	11 (9)
Other Middle Eastern countries	
Iran	5 (5)
Oman	7 (7)
Kuwait	3 (3)
Lebanon	1 (1)
Turkey	1 (1)
Yemen	1 (1)
Countries outside the Middle East	
Tunisia	3 (3)
United Kingdom	3 (3)
Algeria	2 (2)
France	2 (2)
Netherlands	2 (2)
Austria	1 (1)
Italy	1 (1)
Malaysia	1 (1)
Source and destination country	No. cases exported
KSA	
Algeria	2 (2)
Netherlands	2 (2)
United States	2 (2)
Austria	1 (1)
Greece	1 (1)
Malaysia	1 (1)
United Kingdom	1 (1)
Qatar	
Tunisia	1 (1)
UAE	
France	1 (1)
Jordan	
Italy	1 (1)


*Based on World Health Organization case counts. MERS-CoV, Middle Eastern respiratory syndrome coronavirus; KSA, Kingdom of Saudi Arabia; UAE, United Arab Emirates.

To calculate exportations, we used a simple multiplier model whereby MERS-CoV incidence rates among source country residents were extrapolated to rates among visitors. In particular, for each source country, the expected number of cases among travelers was calculated by multiplying the infection rate among residents in the affected countries (total number of cases divided by number of residents) by the number of travelers and the days travelers spend, on average, in the affected countries (*9*). To calculate incidence rates in destination countries, the number of symptomatic cases was considered to be 10 times greater than the reported number of cases (*8*). Calculations were made by using Excel (Microsoft Corporation, Redmond, Washington, USA). The probability of exportation was calculated while assuming the number of exportations was Poisson distributed, with the mean equal to the expected exportations (Technical Appendix 1, and Technical Appendix 2). 

Expected annual exportations of symptomatic MERS-CoV infection cases among visitors to the Middle East source countries were highest among visitors to KSA (Table 2). For visitors returning from KSA, expected exportations were higher among visitors returning to Algeria, where the number of expected exportations was 1 (95% CI 0–5) and the likelihood of >1 exportation was 58%; and Malaysia, where the number of expected exportations was 1 (95% CI 0–5) and the likelihood of >1 exportation was 47%.

**Table 2:** Estimated annual number of symptomatic cases of MERS-CoV infection exported and likelihood of >1 exportation for countries outside the Middle East among visitors to the source countries*

Country of destination	Observed no. symptomatic cases exported	No. cases exported from source countries (95% CI); % likelihood for >1 exportation†		
		Jordan	KSA	UAE
Algeria	2 from KSA	0 (0–4); 0	1 (0–5); 58	NA
Austria	0	0 (0–4); 0	0 (0–4); 1	NA
France	1 from UAE	0 (0–4); 0	0 (0–4); 11	0 (0–4); 9
Italy	1 from Jordan	0 (0–4); 0	0 (0–4); 3	0 (0–4); 9
Greece	0	0 (0–4); 0	0 (0–4); 1	NA
Netherlands	2 from KSA	0 (0–4); 0	0 (0–4); 5	0 (0–4); 4
Malaysia	1 from KSA	0 (0–4); 0	1 (0–5); 47	NA
Tunisia	1 from Qatar	0 (0–4); 0	0 (0–4); 19	NA
United Kingdom	1 from KSA	0 (0–4); 0	0 (0–4); 24	0 (0–4); 35
United States	0	0 (0–4); 1	0 (0–4); 27	0 (0–4); 31


*The incubation period considered was 5.5 days (95% CI 3.6–10.2 days) (*8*). MERS-CoV, Middle Eastern respiratory syndrome coronavirus; KSA, Kingdom of Saudi Arabia; UAE, United Arab Emirates; NA, not available. †Information on tourists to Qatar was not available.

Expected exportations among residents from the Middle East source countries traveling abroad also were estimated to be higher for visitors from KSA (Table 3). Expected exportations among residents in KSA visiting abroad were highest for the United States, where the number of expected exportations was 1 (95% CI 0–5) and the likelihood of >1 exportation was 51%.

**Table 3:** Estimated annual number of symptomatic cases of MERS-CoV infection exported and likelihood of >1 exportation for countries outside the Middle East among source country residents traveling outside the Middle East*

Country of destination	Observed no. symptomatic cases exported	No. cases exported from source countries (95% CI); % likelihood for >1 exportation†			
		Jordan	KSA	Qatar	UAE
Italy‡	0	0 (0–4); 1	0 (0–4); 18	NA	0 (0–4); 8
Malaysia§	0	0 (0–4); 0	0 (0–4); 33	0 (0–4); 1	0 (0–4); 2
Tunisia‡	0	0 (0–4); 0	0 (0–4); 2	0 (0–4); 0	0 (0–4); 0
United Kingdom§	0	0 (0–4); 0	0 (0–4); 35	0 (0–4); 3	0 (0–4); 25
United States§	2 from KSA	0 (0–4); 0	1 (0–5); 51	0 (0–4); 2	0 (0–4); 8


*The incubation period considered was 5.5 days (95% CI 3.6–10.2 days) (*8*). MERS-CoV, Middle Eastern respiratory syndrome coronavirus; KSA, Kingdom of Saudi Arabia; UAE, United Arab Emirates; NA, not available. †Information on Middle Eastern visitors to France, Netherlands, and Greece was not available. Number of Middle Eastern residents visiting Algeria and Austria was not available by country of residence. ‡Number of Middle Eastern visitors traveling to Italy and Tunisia corresponds to persons whose nationality is that of Middle Eastern source countries. §Number of Middle Eastern visitors traveling to Malaysia, the United Kingdom, and the United States corresponds to residents in the Middle East.

## Conclusions

More complex models have been developed to characterize exportations of infectious disease cases via infectious travelers (*10*,*11*). The main advantage of our approach is its simplicity and reproducibility within short timeframes. This model also complements previous work on the risk for MERS-CoV exportation by global air travel, which attempted to quantify travel volume to the affected areas and calculate exportations among Hajj pilgrims in KSA (*12*,*13*). In our calculations, however, the visitor data included persons traveling by air, land, and sea and distinguished between visitors to the Middle East and persons from the Middle East traveling abroad over the period of 1 year. Unlike Khan et al. (*12*), we accounted for the rate of infection in the source countries by calculating source country–specific MERS-CoV incidence rates. Furthermore, by accounting for the different travel volume of visitors to the Middle East compared with Middle East residents traveling to non–Middle Eastern countries, we suggested the mode of exportation (visitor to the Middle East vs. Middle East resident visiting abroad). For Malaysia, estimated expected exportations were higher among visitors to source countries than among source country residents visiting abroad, consistent with the number of exportations detected by surveillance. However, the opposite was true for the United States, where 2 source country residents were found to have MERS-CoV infection during their US visit (*7*).

Our findings have limitations. The estimates are based on historical incidence data. Thus, they are especially applicable for periods in which the incidence of the disease remains stable. For outbreaks characterized by a highly transmissible pathogen, the model might severely underestimate exportations if incidence rates are not projected to the period for which exportations are calculated. In addition, our small multiplier only took into account exportations of symptomatic cases. Given that most of the detected exportations resulted in hospitalization of the infected case-patient, the number of exportations calculated here likely refers to exportations of severe symptomatic cases. Recent results from a serologic survey performed during December 2012–December 2013 indicate that there might be as many as 44,951 (95% CI 26,971–71,922) persons infected with MERS-CoV in KSA (*14*). During that period, there were 125 reported confirmed cases; thus, the ratio of asymptomatic to reported cases might be much higher, possibly in the hundreds. This finding suggests that exportations of asymptomatic cases might be several orders of magnitude higher than those of severe symptomatic cases.

The upper bound of exportations for the countries least likely to receive symptomatic case importations was 4, suggesting that small numbers of importations are largely stochastic events. Although every country is at risk, as illustrated by the recent outbreak of MERS-CoV infections in South Korea that was triggered by just 1 importation (*15*), our model can be used to assess risk level. Combined with other indicators, our model can help determine the level of additional public health measures (e.g., border screenings) required for infectious diseases threats from abroad.

No exportations of symptomatic cases were detected in some of the top travel volume countries for trips originating in KSA, Jordan, UAE, and Qatar. Travel volume to Egypt, India, and Pakistan was several times higher than that for other countries where MERS-CoV has been detected. However, only 1 case of MERS-CoV infection has been detected in Egypt (in a KSA resident) (http://www.who.int/csr/don/2014_05_01_mers), and no MERS-CoV cases have been detected in India or Pakistan. This finding suggests that MERS-CoV introductions may have already occurred in these countries but have not been detected.

In summary, by adapting a simple model of disease spread, we estimated the expected number of MERS-CoV symptomatic case exportations during a 1-year period among visitors to the Middle East and visitors from KSA, Jordan, Qatar, and UAE to non–Middle Eastern countries where case exportations have occurred. Our estimations suggest that the risk for repeated exportations of severe symptomatic MERS-CoV cases is low, although the number of asymptomatic case exportations might be higher.

Technical Appendix 1 Methods for calculating exportations of cases of Middle East respiratory syndrome coronavirus (MERS-CoV) infection.

Technical Appendix 2 Index on estimating spread of Middle East respiratory syndrome coronavirus (MERS-CoV) infection by travel.
