# Supplementary material for: Exportations of Symptomatic Cases of MERS-CoV Infection to Countries outside the Middle East
Source: Emerg Infect Dis. 2016 Apr;22(4):723–5. doi: 10.3201/eid2204.150976 (PMC4806968; doi:10.3201/eid2204.150976)
Supplement: Technical Appendix 1 — Methods for calculating exportations of cases of Middle East respiratory syndrome coronavirus (MERS-CoV) infection. [file 15-0976-Techapp-s1.pdf]

Article DOI: http://dx.doi.org/10.3201/eid2204.150976

Exportations of Symptomatic Cases of MERS-CoV Infection to Countries outside the Middle East

Technical Appendix 1

Methods

Data

We used data on laboratory-confirmed cases in the source countries, census data, and travel volume. Data regarding the number of cases of infection Middle East respiratory syndrome coronavirus (MERS-CoV) were taken from the case list of the World Health Organization, updated as of January 16, 2015. To calculate the infection rate we divided the number of confirmed cases in each source country of origin with onset date of 2013 through 2015 (as of January 16, 2015) by the total population size of the country of origin and days between January 1, 2013 and January 16, 2015 (the exact date of outbreak onset for each country remains unknown and so a common period with end date close to the date of the last report date was chosen for all source countries). Population estimates were extracted from national publicly available sources (*1*–*4*).

Data on arrivals of visitors to and from the Middle East source countries, per nationality/ country of residence, were extracted from the United Nations World Tourism Organization (UNWTO) Yearbook of Tourism Statistics (*5*); for UAE official tourism statistics were used as well (*6*,*7*). Travel volumes extracted from UNWTO Yearbook include all modes of travel (air, road, sea) and refer to 2012.

For arrivals of visitors to the Middle East, data from UNWTO yearbook were extracted for Saudi Arabia and Jordan. For the United Arab Emirates (UAE), visitor data were only available for Dubai (for 2012) and Abu Dhabi (period: Jan–Apr 2013) for the United Kingdom, the United States, France, Italy, Netherlands; the travel volume for Abu Dhabi was scaled to a

year by multiplying by 3 (*6*,*7*). For Qatar, data on arrivals of nonresident visitors were not available.

Data on arrivals of visitors from the source countries on destination countries were extracted from UNWTO Yearbook of Tourism Statistics. Information on arrivals of Middle East source country visitors to Algeria, Austria, France, the Netherlands, and Greece was not available.

Information on the length of stay of visitors in the Middle Eastern source countries was taken from different sources. For visitors traveling to Jordan and Qatar, data were drawn from the UNWTO Compendium of Tourism Statistics (*8*). For Qatar, data were only available for 2008; for Jordan, 2012 data were used (*8*). For Saudi Arabia, length of stay was obtained per nationality for 2006 from official statistics (*9*). For each nationality, number of passengers per length of stay interval was available. The final length of stay was a weighted average between the midpoints for each interval, in which the weights were the share of travelers in that length of stay category. Lengths of stay larger than 10.2 days were limited to 10.2 days (the upper bound of the incubation period [*10*]), to restrict the estimated number of exportations to infectious cases. If using a longer time, exportations would include number of residents in the Middle East source countries that had MERS-CoV cases but were no longer infectious, less useful to understand risk of person-to-person transmission in the destination countries. For UAE, data were retrieved from Dubai (2012 data) and Abu Dhabi (Jan–Apr 2013) official websites (*6*,*7*). For UAE, length of stay data were not available per nationality and so an average across all international destination countries was used. For source countries residents visiting abroad, duration of exposure in the source countries was taken to be 10.2 days, the upper bound of the incubation period, again to restrict our estimations to arriving MERS-CoV infectious cases (*10*).

Estimation

Overview

For each destination country, we calculated the expected number of exportations, and the probability of exportation from each of the source countries (Saudi Arabia, Jordan, Qatar, UAE) among travelers to and from the source countries. For each source country, the expected number of cases among travelers was calculated by multiplying the infection rate among residents in the affected countries by the person-time at risk among travelers (*11*). The incidence rate of infection

among travelers was estimated by multiplying the travel volume by the average duration of stay of travelers in each source country. To calculate the expected number of symptomatic cases exported to the destination country we further adjusted for under-reporting: expected number of symptomatic cases exported to destination country = ratio of symptomatic to reported cases × [cases in source country)/(total population in source × outbreak duration)] × [(number of travelers) × (traveler's duration of stay)] (*1*).

For MERS-CoV, the ratio between reported and estimated total symptomatic MERS-CoV cases has been calculated to be 10 (*10*). We thus used the factors of 10 in expression (*1*) to calculate the total number of expected annual exportations of symptomatic cases.

In both calculations, we used Poisson confidence intervals to reflect stochastic variation in the expected number of exportations (*11*). CIs do not take into account uncertainty in the number of travelers, which is likely small if compared to uncertainty related to the outbreak size and so will be unlikely to bias the results.

We also calculated the likelihood of disease exportation to any of the countries considered. We assumed that the number of symptomatic cases exported to any destination country followed a Poisson distribution with a mean equal to expression 1. We then estimated the probability of the number of exportations being different and higher than 0, that is, the probability of having at least one exportation.

Exportations among Visitors to Source Countries

To calculate the number of exportations among visitors to the source countries, the number of travelers to the source countries was taken to be the total number of persons traveling to the source countries. This includes groups traveling for different purposes such as tourists, professionals traveling for business, or pilgrims. Travelers' duration of stay in the source countries was taken to be the average length of stay of visitors in each country.

Exportations among Source Country Residents Traveling outside Source Countries

To estimate exportations among travelers who are residents in the Middle Eastern source countries, the number of travelers was considered to be the number of Middle Eastern residents visiting the destination countries. Visitors from the source countries include source country residents (persons that live in the Middle East source countries) and/or persons whose nationality

is that of the source countries (for the United Kingdom, the United States, Malaysia, the number of Middle East residents visiting in these countries was available; for Italy and Tunisia, the number of visitors was available per nationality only). Length of stay in the Middle East was taken to be the upper bound of the incubation period (10.2 days [*10*]), to ensure exportations were restricted to infectious persons.

References

1. GCC Statistical Center. Qatar social statistics 2003–2012 [cited 2015 Dec 29]. http://gccstat.org/en/elibrary/publications/qatar/item/qatar-social-statistics-2003-2012

2. Jordan Department of Statistics. Jordan statistical yearbook 2012. Amman, Jordan: Jordan Department of Statistics; 2012.

3. United Arab Emirates National Bureau of Statistics. Population estimates 2006–2010 [cited 2016 Jan 4]. http://www.fcsa.gov.ae/EnglishHome/ReportDetailsEnglish/tabid/121/Default.aspx?ItemId=1914&PTID=104&MenuId=1

4. Saudi Arabia Central Department of Statistics and Information. Facts about Kingdom [cited 2016 Jan 4]. http://www.cdsi.gov.sa/english/index.php?option=com_content&view=article&id=56&Itemid=147

5. World Tourism Organization. Yearbook of tourism statistics 2008–2012, 2014 edition [cited 2016 Jan 4]. http://www2.unwto.org/publication/yearbook-tourism-statistics-data-2008-2012-2014-edition

6. Dubai Department of Tourism and Commerce Marketing. Hotel statistics [cited 2016 Jan 4]. http://dubaitourism.ae.php54-1.ord1-1.websitetestlink.com/trade-resources/statistics/hotel-statistics

7. Abu Dhabi Tourism and Culture Authority. Hotel establishment statistics monthly report April and year to date (January–April) [cited 2014 July 1]. http://tcaabudhabi.ae/en/about/Pages/reports-and-statistics.aspx

8. World Tourism Organization. Compendium of tourism statistics 2008–2012, 2014 edition [cited 2016 Jan 4] http://www2.unwto.org/publication/compendium-tourism-statistics-data-2008-2012-2014-edition

9. Saudi Commission for Tourism and Antiquities. Tourism statistical database, 2006 [cited 2014 July 1]. http://tsdb.mas.gov.sa

10. Cauchemez S, Fraser C, Van Kerkhove MD, Donnelly CA, Riley S, Rambaut A, et al. Middle East respiratory syndrome coronavirus: quantification of the extent of the epidemic, surveillance biases, and transmissibility. Lancet Infect Dis. 2014;14:50–6 PubMed http://dx.doi.org/10.1016/S1473-3099(13)70304-9.

11. Fraser C, Donnelly CA, Cauchemez S, Hanage WP, Van Kerkhove MD, Hollingsworth TD, et al.; WHO Rapid Pandemic Assessment Collaboration. Pandemic potential of a strain of influenza A (H1N1): early findings. Science. 2009;324:1557–61 PubMed http://dx.doi.org/10.1126/science.1176062.
